# Global gene expression profile progression in Gaucher disease mouse models

**DOI:** 10.1186/1471-2164-12-20

**Published:** 2011-01-11

**Authors:** You-Hai Xu, Li Jia, Brian Quinn, Matthew Zamzow, Keith Stringer, Bruce Aronow, Ying Sun, Wujuan Zhang, Kenneth DR Setchell, Gregory A Grabowski

**Affiliations:** 1 The Division of Human Genetics, Cincinnati Children's Hospital Research Foundation, Cincinnati, OH 45229-3039, USA; 2 The Division of Pathology and Laboratory Medicine, Cincinnati Children's Hospital Research Foundation, Cincinnati, OH 45229-3039, USA; 3 The Division of Biomedical Informatics, Cincinnati Children's Hospital Research Foundation, Cincinnati, OH 45229-3039, USA

## Abstract

**Background:**

Gaucher disease is caused by defective glucocerebrosidase activity and the consequent accumulation of glucosylceramide. The pathogenic pathways resulting from lipid laden macrophages (Gaucher cells) in visceral organs and their abnormal functions are obscure.

**Results:**

To elucidate this pathogenic pathway, developmental global gene expression analyses were conducted in distinct *Gba1 *point-mutated mice (V394L/V394L and D409 V/null). About 0.9 to 3% of genes had altered expression patterns (≥ ± 1.8 fold change), representing several categories, but particularly macrophage activation and immune response genes. Time course analyses (12 to 28 wk) of INFγ-regulated pro-inflammatory (13) and IL-4-regulated anti-inflammatory (11) cytokine/mediator networks showed tissue differential profiles in the lung and liver of the *Gba1 *mutant mice, implying that the lipid-storage macrophages were not functionally inert. The time course alterations of the INFγ and IL-4 pathways were similar, but varied in degree in these tissues and with the *Gba1 *mutation.

**Conclusions:**

Biochemical and pathological analyses demonstrated direct relationships between the degree of tissue glucosylceramides and the gene expression profile alterations. These analyses implicate IFNγ-regulated pro-inflammatory and IL-4-regulated anti-inflammatory networks in differential disease progression with implications for understanding the Gaucher disease course and pathophysiology.

## Background

Gaucher disease, an autosomal recessive disorder, is a common lysosomal storage disease. Insufficient activity of acid β-glucosidase (glucocerebrosidase, GCase, E.C.3.2.1.45) in all cells leads to the substrate accumulation including glucosylceramide and glucosylsphingosine, and the various clinical phenotypes. The pathologic hallmark of Gaucher disease is the presence of lipid laden macrophages, a.k.a., Gaucher cells, in visceral organs [1]. The macrophages are thought to be the primary visceral cells involved in all variants, and these cells become progressively numerous and engorged with glucosylceramide by phagocytic processes. By yet undefined mechanisms, this process leads to tissue dysfunction that can result in fibrosis and scarring during the later stages of the disease.

Some of these tissue changes have been attributed to "activation" of the engorged macrophages with subsequent release of inflammatory agents. Indeed, some Gaucher disease patients had increased levels of pro-inflammatory (i.e., TNFα, IL-6, IL-8, and IL-1β) and anti-inflammatory cytokines (i.e., CD14) in serum and/or tissues [2-4]. TNFα production has been suggested as a response to glucosylceramide accumulation in Gaucher disease patients [2]. Serum levels of M-CSF, sCD14 (a macrophage activation marker), and IL-8 can also be increased and correlations have been made with the severity of Gaucher disease [3]. An *in situ *study of spleen from a Gaucher disease patient showed increased expression of anti-inflammatory mediators in macrophages, including CCL18, CD163, chitotriosidase, IL-1Ra, and CD14 [5]. Such anti-inflammatory mediators are considered markers of alternatively activated macrophages (aamφ) [6-10] and implicate secreted cytokines as pathophysiological agents in Gaucher disease. Such studies also suggest a central role of glucosylceramide in altered macrophage function as an initiator of the disease pathogenesis.

How the insufficiency of GCase activity and the subsequent metabolic disturbances related to glucosylceramide and other sphingolipids (GSLs) could lead to such inflammatory imbalances remains obscure. However, the consequent imbalances of ceramide, sphingosine, and sphingosine 1-phosphate in Gaucher disease could affect immunologic responses, inflammation and cell proliferation [11-13]. These and other studies implicate profound systematic pathophysiological changes rather than simple lipid accumulation as the basis of the disease [1]. The pathologic manifestations of various organs suggest that the defective glucosylceramide hydrolysis and substrate accumulation in multiple organs affects numerous metabolic networks. Consequently, systematic transcriptome analyses could provide useful insights into the resultant molecular events underlying GCase deficiency and glucosylceramide storage as well as related tissue pathogenesis in Gaucher disease. In addition to visceral processes, some correlations of neuropathologic involvement with gene expression profiles in brains from neuronopathic Gaucher disease patients or *Gba1 *variant mice [14,15] provide isolated cross-sectional views of the disease processes. However, they have not provided insight into the dynamic or sequential nature of such pathophysiological progression.

Here, viable *Gba1 *point-mutated mice, V394L/V394L (4L) and D409 V/null (9 V/null), were used to explore the temporal and spatial profiles of tissue and *Gba1 *mutation-related gene expression by genome-wide mRNA microarrays and immunohistochemical analyses. Particular focus was on macrophage activation (classic and alternative) responses and IFNγ regulated pro-inflammatory or IL-4 regulated anti-inflammatory networks. These studies established a starting point for understanding the basis of the progressive pathophysiology in Gaucher disease at a molecular/tissue level.

## Results

### Phenotype of *Gba1 *V394L homozygotes (4L) and D409 V/null (9 V/null) mice

**Figure 1:**
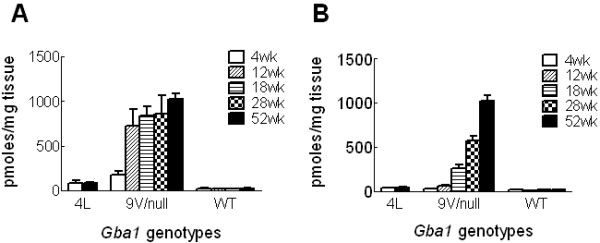
**Glucosylceramide in 9 V/null and 4L mice**. **A**: LC-MS/MS analyses showed an age dependent progressive accumulation of glucosylceramide in the lung of 9 V/null mice: 6-fold, 28- to 33-fold, and 40-fold greater than WT at 4, 12-28, and 52 wk, respectively. In the 4L lung, there was ~3-fold increase by 4 to 52 wk. **B**: Similar, progressive accumulation of liver glucosylceramide: 5-fold, 15-fold, 32-fold, and 46-fold greater than WT at 12, 18, 28, or 52 wk, respectively. Glucosylceramide levels in the liver of 4L were 2-fold increased over the WT.

**Figure 2:**
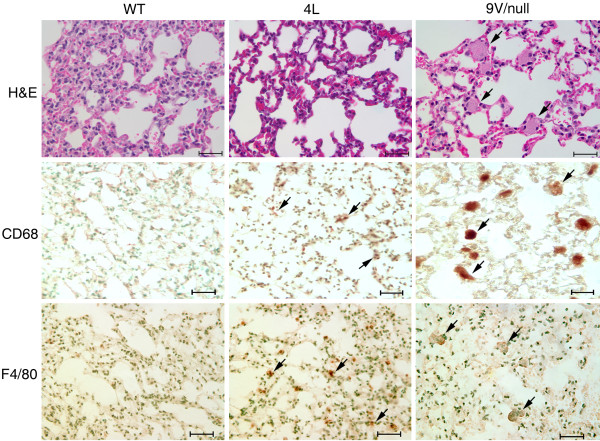
**Histology and immunohistology of lung from WT, 4L, and 9 V/null mice**. Lung sections were from 28-wk age-matched mice. Representative H&E sections showed no storage cells in the alveolar spaces of WT or 4L lungs, whereas abundant large storage cells were present in 9 V/null lung (e.g., arrows). Immunohistochemistry staining with CD68 and F4/80 antibodies showed large positively-staining storage cells in 9 V/null lung (e.g., arrows), i.e., macrophage origin. In comparison, 4L lung had a few, small CD68 and F4/80 positive cells (e.g., arrows). WT lungs did not exhibit positive-staining cells. The scale bars are 20 μm. Magnification = 400×.

4L or 9 V/null mice appear phenotypically normal and have survived ~ 2 years. All mice have grossly normal behavior, reproduce, and show no gross evidence of CNS abnormalities. The residual GCase activity levels in visceral organs were 6-7% or 4-7% of WT in 4L or 9 V/null, respectively [16]. By LC-MS/MS analyses, accumulations of glucosylceramide were detected in 9 V/null lung and liver as early as at 4 wk (2- to 6-fold over the WT controls), and then progressed up to 33-fold during 12 to 28 wk and >40-fold at 52 wk (Figure 1). In 4L mice, glucosylceramide levels in lung and liver were 2- to 3-fold increased at 4 or 52 wk, respectively (Figure 1). Numerous large storage cells were observed in 9 V/null lungs (Figure 2) and to a lesser extent in liver at 12, 18, and 28 wk. Such cells were much smaller and less numerous in 4L lungs (Figure 2). The macrophage nature of storage cells was evidenced by their CD68 and F4/80 positivity (Figure 2).

### Differential gene expression in tissues from Gaucher mice

**Figure 3:**
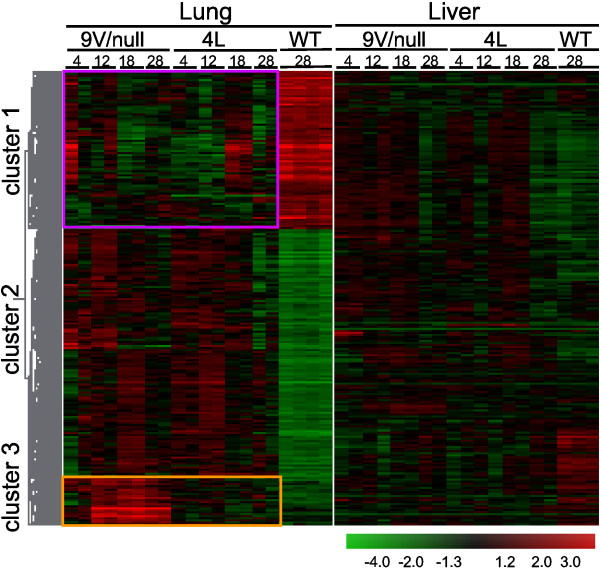
**Hierarchical cluster analyses of differentially expressed genes in lung and liver of *Gba1 *mutant mice**. The two-dimensional hierarchical clustering charts include 1171 genes differentially expressed in 9 V/null lung, and the corresponding genes in 9 V/null liver, and those in 4L or WT lung and liver at ages of 4-, 12-, 18-, or 28-wk (see Materials and Methods). The expression patterns of duplicate chip data are displayed in adjunct lanes for each transcript. The heat map (Pearson correlation) displays gene group correlation patterns across all time points. Each column represents the general expression profile of 1171 genes at a time point. Each row represents a single gene across all time points. The intensities (*red *to *green *scale bar) are the log ratios, log_2_(D/C), where D and C are the gene expression levels in the samples from *Gba1 *mutant mice and WT controls, respectively, and indicate up- or down-regulated RNA expression. Clustering was performed by distance measurements. In cluster 1 are the 407 genes down-regulated in 9 V/null and 4L lung compared to the corresponding genes in WT lung (genes listed in Additional file 2). In cluster 2 are the 647 genes had a variable degrees of up-regulation compared with WT controls. In cluster 3 (orange rectangle) is a group of 117 genes consistently up-regulated in the lung of 9 V/null mice at ages of 12- to 28-wk. These include 40 macrophage activation genes (see Figure 4).

**Figure 4:**
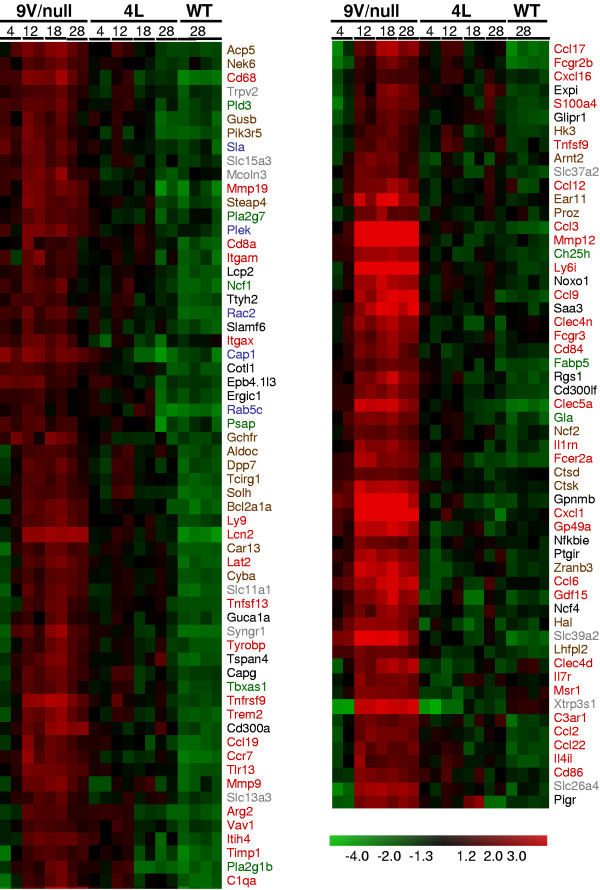
**Group 3 over expressed genes in 9 V/null**. Two-dimensional hierarchical cluster analyses of the 117 genes in cluster 3 (Figure 3) from 9 V/null lung showed high expression at ages of 12-, 18-, or 28-wk compared with the genes in 4L and WT lungs. Among these 117 genes, 40 were macrophage activation genes and included 13 pro-/anti-inflammatory pathway genes. The name of each gene is listed and their major functional categories are presented at the right: macrophage activation related (red), intracellular signaling (blue), lipid metabolic process (green), catalytic activity (brown), transport (grey), and others (black). The expression level of each gene at different time points is coded according to the color scale.

The RNA samples were from lungs and livers of 9 V/null and 4L mice at the ages of 4-, 12-, 18- and 28-wk. The probe sets for each gene in the mutant mice that showed significantly differential expression were compared with those from the strain background matched WT. In these mutant models, the altered probe signals ranged from 560 to 1806 of the 45101 total probes on the chip. For further analyses, the genes were assigned to the probe sets. At a false positive rate (FDR) of 0.01, about 1.2-4% of total genes were significantly differentially expressed: 1171 or 402 genes in lung or liver, respectively, of 9 V/null mice; 1375 or 406 genes were in lung or liver, respectively, of 4L mice (Additional file 1). The differentially expressed genes in each tissue were segregated into 12 major functional groups with statistical significance across the two tissues in both mouse models to provide global profiles of gene expression. The functional categories include catalytic activity, cell death, cytoskeleton, immune response, intracellular signaling cascade, kinase activity, lipid metabolic process, lysosome, macrophage activation, response to stress, transcription, and transport (Table 1 and Additional file 2). In lung, both mouse models showed similar spectra of gene expression patterns for these functional groups, except genes for immune response and macrophage activation that were more highly altered in 9 V/null mice. In 9 V/null and 4L livers, fewer functional groups (6 to 7) were significant in comparison with lung profiles (Table 1). Interestingly, genes for cell death did not reach significance in 4L liver, and genes for immune response were significant only in 9V/null lung (Table 1). The macrophage activation genes were grouped as a functional category, because the macrophage is the hallmark of Gaucher disease, and large numbers of storage cells (macrophages) were observed in the lungs of 12-, 18-, and 28-wk old 9 V/null mice (Figure 2). The enrichment of differentially expressed gene categories was examined by comparing the percentage populating the various gene ontologies in these two mouse models across lung and liver from all time points. Among these 12 different functional categories, the composition of macrophage activation genes in 9 V/null lung was 10% of the total altered genes, and 6% in 4L lung: these represent 1.7-fold enrichment (Table 1). To correlate the gene functional category with their expression profile in *Gba1 *mutant mice, the 1171 significantly differentially expressed genes in 9 V/null lung were analyzed by hierarchical clustering, and three clusters were revealed across all ages in the lung. Cluster 1 showed 407 genes with low expression (35% of total 1171 genes) in 9 V/null and 4L lung across the 4- to 28-wk age range when compared with the WT lung (Figure 3, cluster 1). These down-regulated genes were mainly in the functional categories of catalytic activity, cell death, intracellular signaling cascade, kinase activity, macrophage, and transcription (Additional file 3). Cluster 2 contained about 55% of genes (647 from total 1171 genes) and showed differential degrees of up-regulation in the 9 V/null and 4L lung (Figure 3, cluster 2). Cluster 3 showed 10% of genes (117 from total 1171 genes) with high expression levels exclusively in 9 V/null lung (Figure 3, cluster 3). These same genes did not have distinct patterns in the 9 V/null liver or the 4L lung and liver data sets (Figure 3, right panel). Two-dimensional hierarchical cluster analyses show mutation-type (4L lung vs. 9 V/null lung) and tissue type-related (lung vs. liver) global gene expression profiles. These highly expressed 117 unique genes in 9 V/null lung contained 40 that were macrophage activation genes. The others represented intracellular signaling (5), lipid metabolic process (9), catalytic activity (23), and transport (10) (Figure 4). These initial findings strongly indicated the involvement of macrophage activation genes in Gaucher disease and led to the focus on the expression profiles of macrophage activation related genes in 9 V/null lung.

**Table 1:** Classification of significantly expressed genes in tissues of 9 V/null and 4L mice

Categories	Lung						Liver					
	
	9 V/null			4L			9 V/null			4L		
	
	**No**.	%	p-value	**No**.	%	p-value	**No**.	%	p-value	**No**.	%	p-value
catalytic activity	440	38%	1.49E-06	527	39%	4.24E-09	160	40%	3.37E-04	157	39%	2.61E-03
intracellular signaling cascade	136	12%	7.49E-10	166	12%	2.40E-10	50	13%	7.50E-04	46	11%	1.24E-03
kinase activity	87	8%	8.41E-04	98	7%	1.41E-03	34	9%	8.07E-03	31	7%	4.46E-02
lipid metabolic process	66	6%	7.62E-04	73	5%	1.43E-02	37	9%	1.22E-05	30	7%	7.71E-04
macrophage activation	119	10%	4.50E-04	83	6%	3.29E-03	24	6%	5.74E-03	26	6%	6.93E-03
transcription	195	17%	1.44E-03	276	20%	2.33E-08	83	21%	2.50E-03	76	19%	4.17E-03
cell death	75	7%	1.36E-04	89	7%	3.83E-04	27	7%	4.93E-02			
cytoskeleton	78	7%	3.92E-02	87	7%	5.96E-03						
lysosome	29	3%	1.91E-05	27	2%	1.43E-03						
response to stress	79	7%	1.52E-03	91	7%	1.20E-02						
transport	219	19%	2.14E-04	255	19%	8.03E-03						
immune response	54	5%	4.68E-03									

The functional categories with *p*-value < 0.05 were considered significant by Fisher Exact Test.

No. = number of genes

The empty rows were not significant (*p *> 0.05)

### Expression profile of macrophage activation genes

**Figure 5:**
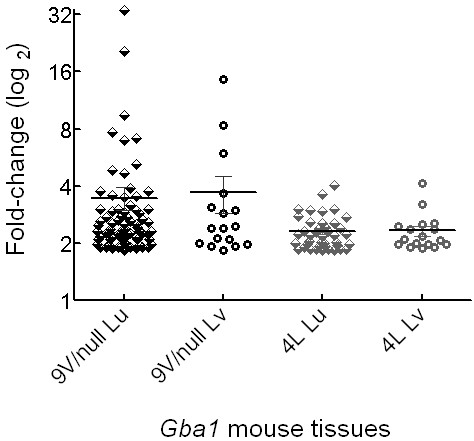
**Consistently up-regulated macrophage activation genes in 9 V/null and 4L mice**. The expression values of each consistently (≥2 time points from 12 to 28 wk) up-regulated macrophage activation genes in the lung (Lu) and liver (Lv) of 9 V/null and 4L mice were plotted on the ordinate. The mean expression values (fold-change) of these genes in each tissue are indicated by a horizontal bar. The numbers of these genes were 96/119 (81%) or 15/21 (71%) in 9 V/null lung or liver, respectively; and 43/83 (52%) or 14/21 (67%) in 4L lung or liver, respectively (Additional file 3). The median fold change for these gene expression values in 9 V/null lung and liver were higher than in the corresponding 4L tissues.

To explore the expression profile of macrophage activation, all 119 such genes (Table 1) were analyzed. At early stages of the disease (4 wk), 44% of these genes (53/119) were significantly altered with 42 up- and 11 down-regulated in the 9 V/null lung (Additional file 4). As the disease progressed (12, 18 or 28 wk), 81% (96/119) of the macrophage activation genes were significantly and consistently up-regulated, and 15% (18/119) of these genes were down regulated in 9 V/null lung (Additional file 4). The genes with expressions significantly changed at 2 or 3 time points from 4 to 28 wk were considered as consistently affected. Similar expression profiles were defined in 4L lung where 88% (73/83) of such genes were altered at 4 wk with 48 up-regulated and 25 down-regulated. From 12 to 28 wk, 84% (70/83) of the macrophage activation genes were affected with 43 up- and 27 down-regulated. The mean fold-change of these consistently up-regulated macrophage activation genes was >3.5-fold in 9 V/null lung and liver, whereas a 2.3-fold change was observed in the corresponding 4L tissues (Figure 5 and Additional file 4).

Using the Ingenuity Analysis System and additional literature sources [5,8], 9 IFNγ-regulated pro-inflammatory and 9 IL-4-regulated anti-inflammatory genes were significantly expressed (fold change ≥ ± 1.8) from 12 to 28 wk (Table 2) in 9 V/null lungs with 5 pro-inflammatory genes significantly altered beginning at 4 wk (Table 2). Most of these genes were cytokines/chemokines or their receptors, including CCL2, CCL3, CCL6, CCL9, CCL19, CXCL1, and CXCL12. Other genes included CD44 (a transmembrane glycoprotein related to cellular adhesion and migration) and the macrophage scavenger receptor 1 (Msr1). Several pro-inflammatory genes [IFNγ, interleukin 6 (IL-6), inducible nitric oxide synthase (NOS2) and TNFα] did not meet the statistical significance (**Bold **genes in Table 2). To evaluate these genes, immunohistochemical analyses were conducted and the lipid-laden macrophages showed antibody positive signals (see below). The results show high expression of these four cytokine proteins in macrophages even though their respective RNA levels were relatively unchanged. Additional immunohistochemical analyses showed consistent positivity with antibodies to CCL2, CCL3, and CCL9; this was concordant with their RNA expression. Similar mRNA analyses showed that 6% of altered genes were in the macrophage activation category in 9 V/null liver or 4L lung and liver. However, none were pro-inflammatory genes (Table 2 and Additional file 5). These data support disease progression and tissue-type related profiles of pro-inflammatory gene expression in Gaucher disease.

**Table 2:** Expression of pro- and anti-inflammatory macrophage activation genes in lungs of 9 V/null and 4L mice

			9 V/null				4L			
**Pathways**	**Symbol**	**Gene Name**	**lu_4 wk**	**lu_12 wk**	**lu_18 wk**	**lu_28 wk**	**lu_4 wk**	**lu_12 wk**	**lu_18 wk**	**lu_28 wk**

IFNγ regulated	IFNγ*	interferon gamma	1.124	1.292	1.336	1.204	1.513	1.278	1.329	1.435
	CCL19	chemokine (C-C motif) ligand 19	1.325	**3.288**	**3.336**	**2.039**	1.738	1.469	1.166	1.536
	CCL2*	chemokine (C-C motif) ligand 2	1.134	**2.835**	**2.564**	**2.467**	1.284	1.220	1.203	1.463
	CCL3*	chemokine (C-C motif) ligand 3	**1.855**	**19.86**	**37.518**	**22.298**	1.464	1.673	1.155	1.347
	CCL6	chemokine (C-C motif) ligand 6	**2.004**	**4.073**	**6.557**	**6.138**	-1.380	-1.090	-1.344	1.228
	CCL9*	chemokine (C-C motif) ligand 9	**1.893**	**5.817**	**10.346**	**9.722**	1.317	1.546	1.512	1.365
	CD44	CD44 antigen	**3.176**	**4.971**	**2.155**	**2.387**	1.060	1.156	-1.045	1.294
	CXCL1	chemokine (C-X-C motif) ligand 1	1.466	**8.56**	**10.786**	**7.739**	1.322	1.252	-1.183	1.663
	CXCL12	chemokine (C-X-C motif) ligand 12	**2.65**	**2.991**	**1.923**	**1.874**	1.679	1.334	1.130	1.030
	IL-6*	interleukin 6	-1.249	1.189	1.029	-1.009	-1.080	-1.261	-1.262	-1.106
	Msr1	macrophage scavenger receptor 1	1.092	**2.843**	**2.808**	**2.322**	1.349	1.515	-1.089	1.076
	NOS2*	nitric oxide synthase 2, inducible, macrophage	1.226	1.207	-1.001	1.13	1.206	1.351	-1.001	1.200
	TNF*	tumor necrosis factor	-1.096	1.036	1.279	1.337	1.184	1.384	-1.008	1.451

IL-4 regulated	IL-4*	interleukin 4	-1.074	-1.007	1.135	1.253	-1.071	1.286	1.107	1.367
	Arg2	arginase type II	1.315	**2.661**	**3.044**	**2.034**	1.787	2.179	1.298	1.344
	CCL17	chemokine (C-C motif) ligand 17	1.146	**3.756**	**8.523**	**5.903**	**1.982**	**2.494**	1.522	1.674
	CCL22	chemokine (C-C motif) ligand 22	1.237	**3.066**	**2.734**	**2.725**	1.402	1.477	1.096	1.559
	CD163*	CD163 antigen	-1.029	1.013	-1.446	-1.391	1.173	1.142	1.234	-1.335
	Igh-6	Immunoglobulin heavy chain 6	**3.036**	**3.066**	**3.430**	**2.565**	**2.864**	**1.810**	1.724	1.637
	IL-1rn	interleukin 1 receptor antagonist	1.417	**3.615**	**3.815**	**2.762**	1.296	1.317	1.376	1.141
	MMP12*	matrix metallopeptidase 12	1.705	**37.346**	**56.278**	**39.721**	1.225	1.023	-1.240	1.217
	MMP19	matrix metallopeptidase 19	1.762	**3.607**	**3.428**	**3.112**	1.659	1.650	1.563	-1.249
	MMP9	matrix metallopeptidase 9	1.272	**2.837**	**4.307**	**2.308**	-1.353	1.233	1.199	1.226
	Retnlα	resistin like alpha	**1.920**	**2.229**	**2.480**	**3.800**	**2.796**	1.538	**2.536**	**1.840**

Note: The gene symbols in **Bold **were not significantly expressed in microarray analysis, but were positive on immunohistochemstry in 9 V/null lung. The numbers show Fold Change in gene expression. The numbers in **Bold **were significantly affected and satisfied with FDR (≤0.01) and Fold Change at ≥ ± 1.8. Genes with * sign were positive on immunohistochemistry. wk = week.

In the IL-4 regulated pathway, 9 anti-inflammatory genes were up regulated in 9 V/null lung (Table 2). Two genes (Igh-6 and Rentlα) were up regulated by 4 wk and then continuously up regulated at 12 to 28 wk. The other seven anti-inflammatory genes were unchanged at 4 wk. These less significant changes in the anti-inflammatory genes at the age of 4-wk reflect the underdevelopment of alternatively activated macrophage features. With age (12-, 18- or 28-wk), all anti-inflammatory genes were consistently up regulated including the NOS2 counteracting enzyme arginase type II (Arg2), cytokine/chemokine CCL17/CCL22, immunoglobulin heavy chain 6 (Igh-6), IL-1 receptor antagonist (IL-1rn), and matrix metallopeptidase MMP9/12/19 (Table 2). Among these genes, MMP12 expression was exceptionally high (37- to 56-fold increased). MMP-12, macrophage elastase, functions to degrade extracellular matrix components, e.g., elastin, and is involved in acute and chronic pulmonary inflammatory diseases associated with an intense airway remodeling [17,18]. The anti-inflammatory genes, e.g., IL-4, and the macrophage scavenger receptor CD163 did not meet statistical significance, but were positive by immunohistochemistry. With 9 V/null liver mRNA, only three anti-inflammatory genes (Igh-6, IL-1rn and MMP12) were consistently up regulated (Additional file 5) indicating differential effects on the IL-4-regulated anti-inflammatory pathway genes in lung and liver. In 9 V/null lung, these data show that the IL-4 regulated anti-inflammatory cytokines/mediators were also temporally and spatially altered and they were expressed in parallel with coordinate pro-inflammatory genes with disease progression.

**Figure 6:**
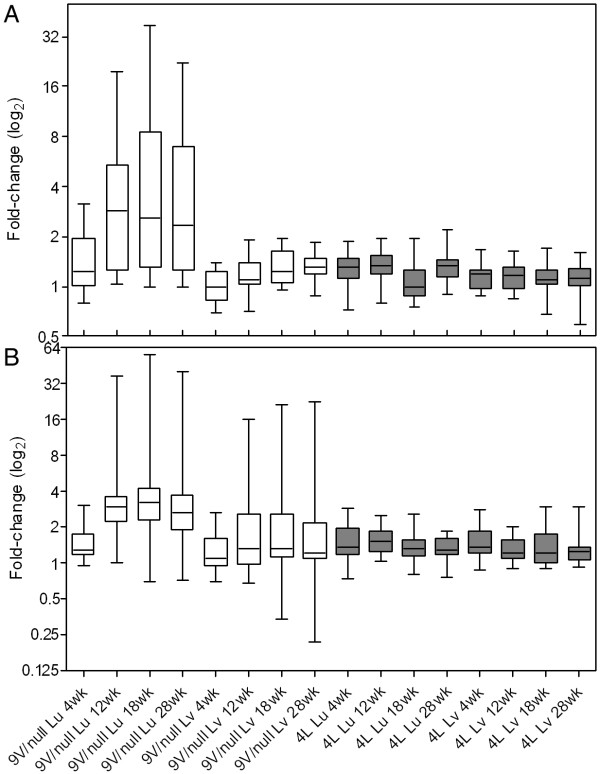
**Time course of expression profiles of pro-inflammatory (A) and anti-inflammatory (B) genes in 9 V/null (white) or 4L (grey) mice**. The box plots of fold change (log2) show the median value of fold change of genes in each group (horizontal bars). The upper and lower boundaries of the boxes indicate the 75^th ^and 25^th ^percentile. The whiskers indicate the minimum and maximum of data set. The median fold change in the pro-(A) and anti-(B) inflammatory genes of 9 V/null lung showed a steep increase from 4 to 12 to 28 wk that paralleled those in 9 V/null livers. The expression levels of these same genes in 4L lung and livers were lower and nearly constant over the time course of the analyses.

The pro- and anti-inflammatory gene expression patterns were analyzed in the more attenuated 4L model. In 4L lung, the percentage of altered macrophage activation genes was ~50% of that in 9 V/null lungs (Table 1). No pro-inflammatory genes were altered in 4L lungs during disease progression (Table 2), and the immunohistochemistry as well as routine histology were identical to WT. Only three anti-inflammatory genes (CCL17, Igh-6 and Rentlα) were consistently (≥2 time points) up regulated in 4L lung. The expression patterns of anti-inflammatory genes in liver of 4L mice were similar to those in the 9 V/null mice. There were no pro-inflammatory genes significantly altered and only the anti-inflammatory genes, Igh-6, IL-1rn, or MMP12, were consistently up regulated in 9 V/null and 4L liver (Additional file 5). In summary, the median expression values of pro-inflammatory and anti-inflammatory genes in 9 V/null lung were 2-fold higher than that in 9 V/null liver and 4L tissues (Figure 6). These data demonstrate the differential RNA expression of pro-inflammatory or anti-inflammatory cytokines/mediators and their correlation with type of mutations and tissues, and disease progression. The results also indicate a differential molecular pathophysiology in 9 V/null or 4L Gaucher disease mice that is likely related to the degree of storage cell infiltration and accumulation of glucosylceramide.

### Commonality of significantly expressed genes involved in macrophage activation

**Figure 7:**
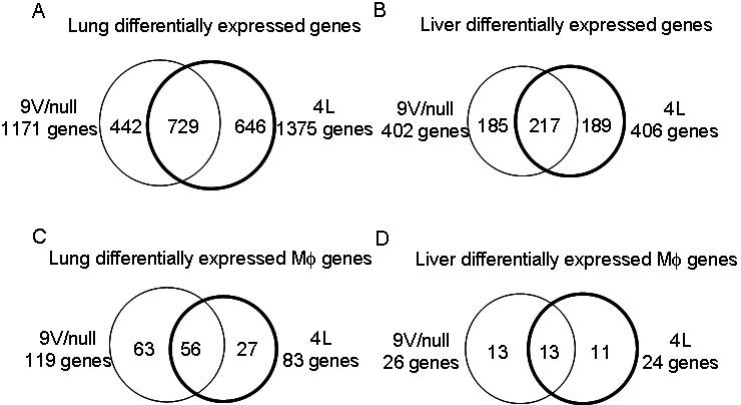
**The commonality and uniqueness of significantly differentially expressed genes (A and B) and macrophage activation genes (C and D) in lungs (A and C) and livers (B and D) from 9 V/null and 4L mice**. Venn diagrams show the absolute number of differentially expressed genes in 9 V/null or 4L tissues. Among the differentially expressed genes, 729 in lung (A) and 217 in liver (B) were common to 9 V/null and 4L mice. In comparison, the uniquely expressed genes in 9 V/null or 4L lungs were 442 or 646, respectively; and 185 or 189 in 9 V/null or 4L livers. In (C and D) the Venn diagrams represent the number of differentially expressed macrophage activation genes in 9 V/null and 4L lungs (C) and livers (D). Among these genes, 56 in lungs and 13 in livers were found common to 9 V/null and 4L mice. The numbers of uniquely expressed genes in 9 V/null or 4L lungs were 63 or 27, respectively; 13 or 11 in 9 V/null or 4L livers. The expression values of these genes are in Additional file 4.

To explore the commonality of affected global or macrophage activation genes between two tissues in *Gba1 *variant mice, comparative analyses were displayed as Venn diagrams. More than 50% of the altered genes globally in lung or liver were concordantly and significantly expressed in 9 V/null and 4L mice (Figure 7A and 7B, Additional file 5). These common genes were distributed in all 12 functional categories. Similar results were obtained by commonality analysis of significantly expressed macrophage activation genes between tissues of both *Gba1 *mutant models. As shown, ~50% of macrophage activation genes in lung or liver of 9V/null mice were similarly affected in 4L mice (Figure 7C and 7D). The intersection of macrophage activation genes in lung or liver from 9 V/null and 4L mice includes pro-/anti-inflammatory cytokines, cytokine receptors, growth factors, and MHC II molecules (Additional file 5). All the matched macrophage activation genes in the intersection were concordantly up or down regulated in both lung and liver of 9 V/null and 4L mice. These data suggest a common basic pathway of macrophage activation genes in multiple tissues of *Gba1 *mutated mice. In the lung of the 9 V/null and 4L models, all genes for antigen processing related to MHC II and some complement components were up-regulated (Additional file 5). The effect of MHC on cytokine production has been shown to be particularly important in resistance of mice to intracellular pathogens [19]. These data imply that GCase deficiency and resultant substrate accumulation stimulated multiple immune response genes. The above analyses demonstrate that substantial numbers of macrophage activation genes were significantly affected in *Gba1 *variant mice, most of them were up-regulated and concordant with the disease progression from 12 to 28 wk, many were shared among tissues, but some were more tissue specific. Although the molecular cascades in macrophage activation are not yet known, the tissue-/mutation-type and disease progress correlated profiles of macrophage activation-related genes suggest common temporally and spatially regulated mechanisms in the pathophysiology of Gaucher disease. The expression profiles of these genes also indicated macrophage activation in *Gba1 *variant mice with significant heterogeneity between the tissues and mutation types.

### Effects of genetic background on mRNA profiles

The genetic backgrounds of 9 V/null (FVB and C57BL/6J-129SvEvBrd) and 4L (C57BL/6J-129SvEvBrd) were slightly different, since the 9 V/null was generated by crosses of 9 V/9 V (C57BL/6J-129SvEvBrd) with null/WT (FVB) mice. To validate the macrophage gene expression profile and evaluate effects of the FVB strain on gene expression, FVB WT data sets (2 chips at 4 different time points each tissue) were run using the same methods and standards. A total of 48 chip sets (each 8 chip data from 9 V/null lung and liver, 4L lung and liver, and FVB WT lung and liver) were loaded into Partek Genomics Suite 6.4, and the data were normalized and analyzed (see Methods). FDR was set at 0.01 and fold change was set at ±1.8 for significance.

The numbers of significantly expressed genes in 9 V/null lung based upon the WT controls from these two different genetic backgrounds are as follows. With the FVB WT as control data set, 910 genes with significantly altered expression were selected. Among them, 10% (90/910 genes) were macrophage activation genes and 1.6% (15/910) were pro-/anti-inflammatory genes (Additional file 6 and Additional file 7 Table S1). The commonality of macrophage genes selected based upon different genetic background was also analyzed. Among 90 macrophage genes (FVB_8chip_), 52% (47 genes) were the same as with genetic matched controls (FVB/C57BL/6J-129Sv_chip_). Also, 83% of INFγ/IL-4 regulated pathway genes were shown to have similar gene expression profiles using either of the two genetic backgrounds. Although some variation exists in the selected genes and their expression significance, the macrophage activation genes, including INFγ and IL-4 pathway genes, were identified as a major functional group using either strains.

### Validation of selected mRNA expression

**Figure 8:**
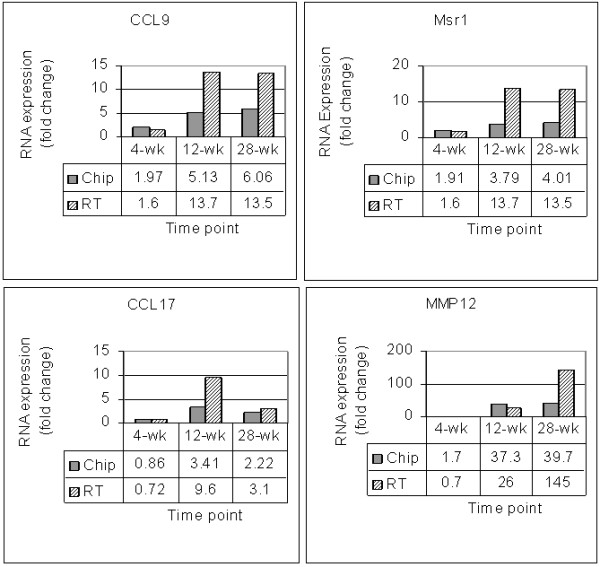
**RT-PCR verification of selected cytokine gene expression levels**. Pooled lung RNA samples used for microarray analyses were used for RT-PCR of CCL9, Msr1, CCL17, MMP12, and β-actin. Signals for these genes from 9 V/null and WT lung RNAs were normalized to β-actin RNA. The average values (fold changes) for RT-PCR (hatched bar) represent the ratios of 9 V/null and WT at each time point (X-axis). The fold change values were from 2 separate triplicate experiments. The comparative fold changes (Table 1) for each respective gene from the microarray analyses are shown as grey bar.

The genes identified by microarray analyses were selectively validated by real-time RT-PCR of the same pooled RNA as used for chip analyses. Primer sets for CCL9, Msr1, CCL17, and MMP12 were used in duplicate assays and β-actin RNA was used as an internal control. The results show similar patterns of cytokine gene expression to those obtained by chip analyses. The fold-change values obtained with real-time RT-PCR were 2- to 4-fold higher than those from chip analyses (Figure 8). This reflects inherent differences of the two methods.

### Immunohistochemical studies of the macrophage activation related genes

Histological and immunohistochemical studies were conducted with lung and liver sections from 9 V/null and 4L mice to correlate gene expression patterns with protein levels. As shown, numerous large storage cells were observed in 9 V/null lung with positivity for the surface antigens, CD68 and F4/80 (Figure 2). CD68 RNA also was elevated in lungs (3.3-7.2 fold) and livers (2.0-4.8 fold) of 9 V/null mice at 12 to 28 wk, but they had no significant alteration in 4L lung and liver (Additional files 1 and 4). This result would be expected because of the large number of storage cells in the lungs of 9 V/null mice. Immunohistochemical staining with CD68 antibody showed high intensity CD68-positive storage cells in the lung (Figure 2, middle) and liver (not shown). CD68-positive cells were quantified and showed 205 and 241 per 20 representative fields (40×) in the lung from two 9 V/null mice. A few small sized CD68-positive cells were observed in WT lung at 28 wk (Figure 2, middle). In 4L lungs, multiple (268 ± 38, 40× field, n = 10) small CD68-positive macrophages were present at 28 wk (Figure 2, middle). F4/80 (Emr1) RNA signals were within ±1.8-fold range in all 9 V/null and 4L tissues. By immunohistochemistry, F4/80 positive macrophages were 190 and 174 per 20 fields (40×) in the lung of two 9 V/null mice. The qualitative staining intensity of F4/80-positive storage cells was weak to medium compared to that of CD68 positive cells in lung of 9 V/null mice (Figure 2, bottom).

**Figure 9:**
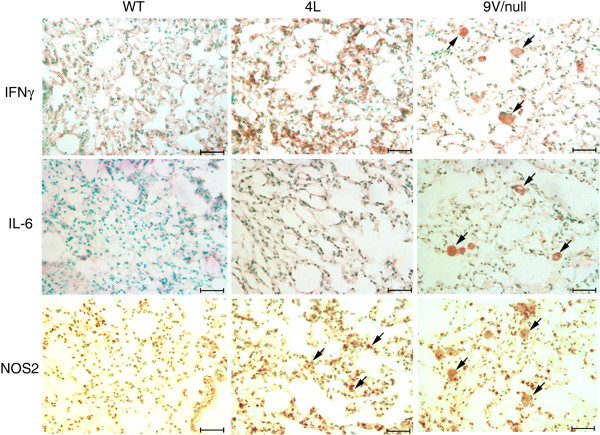
**Expression of IFNg and pro-inflammatory cytokines in lung macrophages of WT, 4L, and 9 V/null mice**. Lung sections were from 28-wk age-matched mice. Large storage cells in 9 V/null lungs were positive for IFNg, IL-6, and NOS2 (arrows). In 4L lung, a few, small NOS2 signal positive cells present (arrows). WT lungs did not exhibit positive staining. The scale bars are 20 μm. Magnification = 400×.

**Figure 10:**
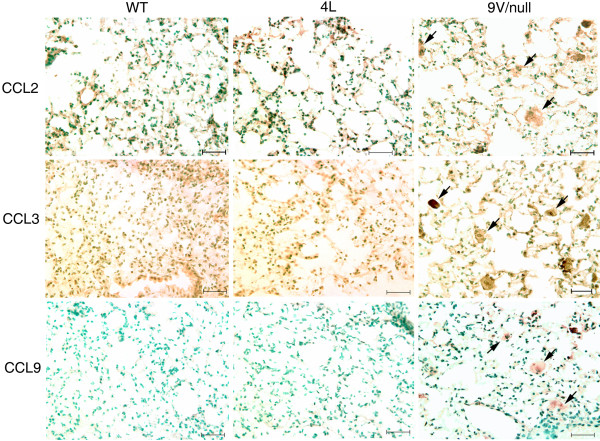
**Expression of CCL2, 3, and 9 in lung macrophages of WT, 4L and 9 V/null mice**. Lung sections from WT (left), 4L (middle) and 9 V/null (right) mice at the age of 28-wk were the same as in Figure 2 and 9. Abundant large storage cells in 9 V/null lungs were positive for CCL2, CCL3, and CCL9 (e.g., arrows). 4L and WT lungs were negative. The scale bars are 20 μm. Magnification = 400×.

**Figure 11:**
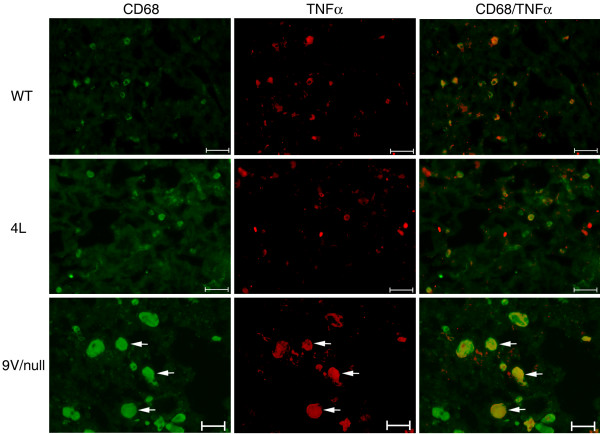
**Expression of TNFα and colocalization with CD68 in lung macrophages from WT, 4L, and 9 V/null mice**. Anti-CD68 is green (FITC) and anti-TNFα is red (Alexa-610). Abundant large storage cells in 9 V/null lungs were CD68 or TNFα positive (e.g., arrows). 4L and WT lungs showed a few scattered small positive cells but none appeared to be positive for both antibodies. TNFα and CD68 colocalized in many of 9 V/null macrophages (yellow). The scale bars are 20 μm. Magnification = 400×.

Macrophage activation in 4L and 9 V/null mice was evaluated using antibodies to cytokines corresponding to genes with differential RNA expression. Antibodies to pro-inflammatory cytokines INFγ, IL-6, NOS2 (Figure 9), CCL2, CCL3, CCL9 (Figure 10), and TNFα (Figure 11) stained storage cells intensely in lungs from 9 V/null mice and were quantified microscopically (see below). Some NOS2 positive cells were present in 4L lungs, but the antibodies to the other cytokines were negative (Figure 9). The RNA expression level of INFγ, IL-6, NOS2 and TNFα in 9 V/null lungs did not satisfy the FDR ≤ 0.01, but the respective proteins could be detected by immunohistochemistry at 12 to 28 wk. By dual immunofluorescence staining, many TNFα signals co-localized with CD68 signals in 9 V/null lung; the TNFα intensity was relatively weak compared with CD68 signal (Figure 11, merged pictures). TNFα signals in 4L lung at 12 and 18 wk were also positive, but with lower intensities and in fewer cells compared with those in 9 V/null lung at 28 wk (data not shown).

**Figure 12:**
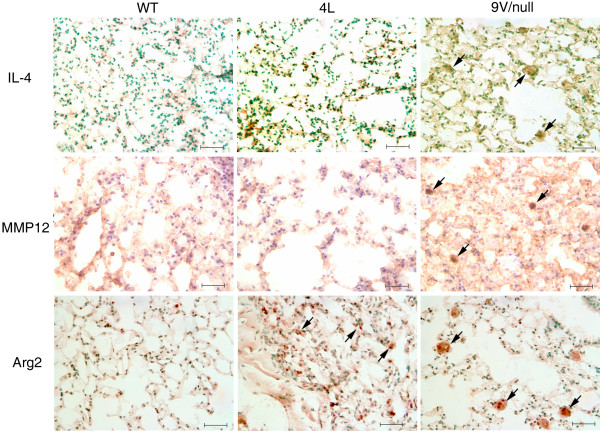
**Expression of IL-4, MMP12 and Arg2 in lung macrophages from WT, 4L, and 9 V/null mice**. Lung sections were as in Figure 2, 9, 10 and 11. Large storage cells in 9 V/null lungs were positive for IL-4, MMP12, and Arg2 (e.g., arrows), whereas only a few were in 4L lungs. WT lungs were negative. The scale bars are 20 μm. Magnification = 400×.

**Figure 13:**
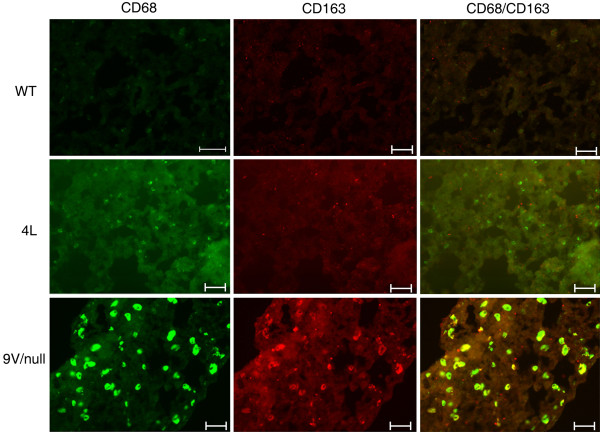
**Expression of the scavenger receptor, CD163, and CD68 in lung macrophages from WT, 4L, and 9 V/null mice**. Anti-CD68 is green (FITC) and anti-CD163 is red (Alexa-610). The colocalization (yellow) of CD163 signals with CD68 in 9 V/null lungs showed about half the storage cells were double positive and no cells staining only with CD163. Some low level signals for CD68 and CD163 were seen in small cells in 4L lungs. The scale bars are 20 μm. Magnification = 400×.

**Figure 14:**
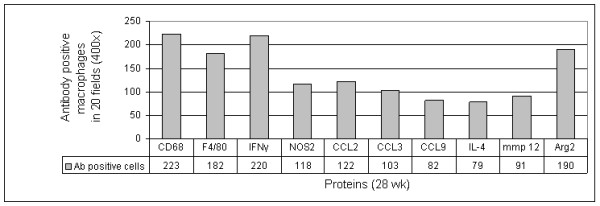
**Heterogeneity of cytokine expression in lung macrophages of 9 V/null mice**. The pro-/anti-inflammatory cytokine antibody stained lung sections from 9 V/null mice of 28-wk (Figure 9, 10 and 12) were analyzed (20 serial fields at 400×). The antibody positive macrophages were quantified in each field, and plotted (Y-axis) for each cytokine protein (X-axis). The numbers below each column are the respective antibody positive macrophages counts.

In 9 V/null lungs, positive signals were also evident for the anti-inflammatory cytokines, IL-4, MMP12, and Arg2 (Figure 12), and CD163 (Figure 13). As with some pro-inflammatory genes, the RNA expression of IL-4 or CD163 genes in 9 V/null lungs did not satisfy the FDR 0.01, but their protein levels were assessed by immunohistochemistry or immunofluorescence staining at 12 to 28 wk. The signals of IL-4 positive macrophages were relatively weak in lung of 9 V/null at 28 wk, and no signals were detected in WT and 4L lung (Figure 12). The staining intensity with Arg2 and MMP12 antibody was at intermediate levels in 9 V/null lung at 28 wk (Figure 12). Arg2 positive cells also were observed in the lung of 4L mice at 28 wk. CD163 signals were significant in lung of 9 V/null mice compared with the signals in WT and 4L tissues (Figure 13), and these mostly co-localized with CD68 signals. In 4L mouse lung, the macrophages were small. The antibody positive macrophages for CCL2, CCL3, CCL9, IL-6, NOS_2_, IL-4, or MMP12 were quantified as above and showed 79 to 122 per 20 fields in 9 V/null lung (Figure 14); INFγ or Arg2 antibody positive cells numbered 220 or 190 per 20 fields. The intensity of each antibody staining at 4 to 28 wk showed a concordant pattern with the RNA expression profile. These studies show differential expression of macrophage activation related genes in the various mice with *Gba1 *mutations that were nearly cell specific.

### IFNγ and IL-4 regulated pathways and gene expression networks

**Figure 15:**
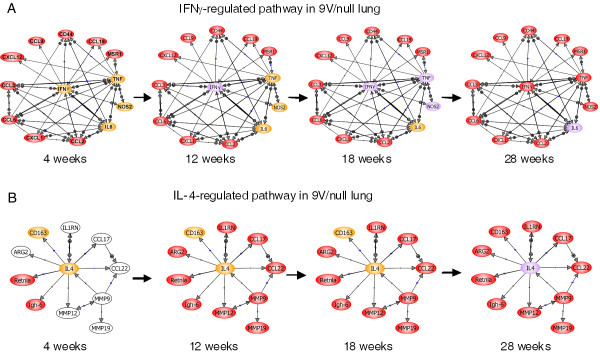
**Time course changes in cytokine network pathways of lungs from 9 V/null mice**. Thirteen pro-inflammatory (A) and eleven anti-inflammatory (B) genes were differentially regulated in lungs of 9 V/null mice across the 4 to 28 wk period. IFNg-regulated pro- or IL-4-regulated anti-inflammatory genes were selected using Ingenuity Pathway Analysis and the pathways were built using PathwayArchitect 2.0.1. Expression levels of pro- or anti-inflammatory genes are represented by color: red ≥ 1.8 fold change; white within ±1.8 fold change. The connecting lines between genes indicate interactions. The expression of IFNγ, IL-6, NOS2, TNFα, IL-4 and CD163 did not meet the statistical significance, but did have antibody positivity. Theses are designated as: weak (orange), medium (purple), or high (red) immunohistological signals.

PathwayArchitect 2.0.1 (Stratagene, La Jolla, CA) and Ingenuity Pathways Analysis (Ingenuity Systems, Inc.) were conducted to summarize the potential interactions of pro-inflammatory or anti-inflammatory cytokines/mediators based on the RNA microarray data and immunohistochemical data (Figure 15). Nine of 13 macrophage pro-inflammatory genes were significantly affected at 4 wk (Figure 15A, left, red or orange color). Beginning at 12 wk, all 13 pro-inflammatory genes were significantly up regulated at the RNA or protein levels, and then remained at up-regulation until 28 wk (Figure 15A). Most of anti-inflammatory genes in the 9 V/null lung were not changed at 4 wk (Figure 15B, white color), but were at 12 to 28 wk (Figure 15B, red or orange color). Interestingly, the up-regulation of IL-4 was only detected at 28 wk. This pro- or anti-inflammatory pathway model summarizes a significant coordination and interaction of pro- or anti-inflammatory cytokines/mediators in the disease process.

**Figure 16:**
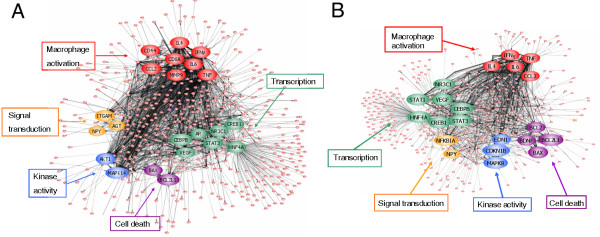
**Global gene expression network profiles**. In 9 V/null lung 391 genes (A) and in 4L lung 345 genes (B) were analyzed as a cascade network using PathwayArchitect 2.0.1 and Ingenuity Pathways Analysis programs. These genes had a high confidence index of direct and indirect interaction with IFNγ/IL-4 macrophage activation genes, which function as core genes (red) and connect to the cascade network via key genes. Gene interactions are shown by the connecting lines. The key genes are those that can be connected to multiple genes. The major key genes are highlighted with different colors for macrophage activation (red), transcription (green), kinase activity (blue), cell death (purple), and signal transduction (yellow) (Additional file 8).

A potential global gene expression network and a cascade regulation initiated by macrophage activation genes were evaluated using PathwayArchitect 2.0.1 and Ingenuity Pathways Analysis programs. A total 391 significantly expressed genes in 9 V/null and 345 genes in 4L lungs were connected in the IFNγ- or IL-4-regulated pro-/anti-inflammatory pathway (Figure 16A and 16B). This network was built with a high confidence index of direct and indirect interactions. In this cascade network, IFNγ- and IL-4-regulated pro-/anti-inflammatory genes as core genes (red) connected with many different functional pathways through key genes. These key genes are significantly connected to the next group genes in the cascade of network (connectivity >1000) [20]. The included main gene groups in each cascade regulation were for transcription (green), kinase activity (blue), cell death (purple), and signal transduction (yellow) (Additional file 8).

## Discussion

The genome-wide transcriptome data presented here demonstrate genotype and tissue-type related gene expression patterns in 9 V/null and 4L mice that are analogues of human Gaucher disease. Interestingly, the percentage of functional categories containing dysregulated RNAs was similar across all 9 V/null or 4L tissues, except for the macrophage activation genes that showed ~2-fold enrichment in 9 V/null lung, the most extensively involved organ (Table 1). Another identified group was that for immune response genes, which were altered only in 9 V/null lung. Among the 54 immune response genes, 47 overlapped with the macrophage group. Since histological analyses did not find obvious T-cell and B-cell infiltration in 9 V/null lung, these immune responses likely emanated from macrophages. Macrophage activation in 9 V/null lung was also supported by hierarchical clustering analysis in which the 117 genes in cluster 3 (Figure 3) were exclusively expressed in 9 V/null, rather than in 4L lung; a third of these were macrophage activation genes (Figure 3, highlighted region). These results indicate significant involvement of macrophage activation genes in 9 V/null lung, which was concordant with the extensive macrophage infiltration in the lungs. Moreover, the complementary histopathological studies showed that the nature of macrophage activation was not uniform between tissues or within macrophages in a specific tissue. These data support the concept of a variety of dysregulated macrophages that are tissue and disease state dependent as a dynamic component of the Gaucher disease/glucosylceramide storage pathophysiology.

Engorged macrophages are a hallmark of Gaucher disease, and these cells were differentially activated in tissues of 9 V/null and 4L mice at the mRNA, cellular, and immunohistochemical levels. The progressively increasing sizes and numbers of macrophages in visceral organs of *Gba1 *variant mice, because of the lipid storage, was particularly evident in lungs of 9 V/null mice from 12 to 28 wk (Figure 1). Immunohistochemistry with the antibodies to selected IFNγ-regulated pro-inflammatory and IL-4-regulated anti-inflammatory cytokines showed that these cytokines were expressed in lung macrophages, but not in lung epithelial cells, i.e., there were pro- and anti-inflammatory responses (or activation) of the lipid-laden macrophage. Also, such analyses showed significant expression of the IFNγ protein in lung macrophages of 9 V/null mice at 12 and 28 wk (Figure 9), but lesser expression in liver. These expression patterns were concordant with the differential activation of macrophages in these tissues as was evident by both microarray and morphologic data. In addition, IFNγ and its regulated pathway genes were less aberrant in tissues of the 4L mice, a more attenuated model, as ascertained by RNA or protein analyses. These results implicate pro-inflammation as a primary pathophysiological mechanism in Gaucher disease, as well as the degree of alterations in this pathway in the severity of Gaucher disease involvement. The production and secretion of cytokines from storage macrophages can be the important factors for the extracellular matrix components and function as they influence the interaction of surrounding macrophages with phagocytotic or endocytotic ligands and propagate the pathophysiology [21].

IFNγ is a potent activator of macrophages and induces the expression of >300 genes, including those in the inflammatory mediator/chemokine pathway [22]. Also, activated macrophages can be a significant source of IFNγ [23-25]. Importantly, IFNγ inhibits macrophage proliferation and protects them from apoptosis [26], thereby prolonging their survival within inflammatory loci [27]. Here, the RNA expression levels of IFNγ, IL-6, NOS2, and TNFα were not significantly increased (FDR = 0.01), but their protein levels were specifically and highly expressed in the large lung macrophages. This observation indicates that the expression of these proteins and RNAs could be missed in whole tissue homogenates in which there may be large dilutional effects, if expression is restricted to specific cell types that make up a small percentage of total cells, e.g., macrophages. Importantly, 12 other cytokines are in the IFNγ pathway and could be downstream modulated by this cytokine. For example, IFNγ can induce TNFα and NOS2, and has differential effects on several individual chemokine genes [8] that provide for selective stimulus sensitivity in mediating restricted pattern of chemokine gene expression. Transcripts for CXCL1/12 and the CCL chemokines (CCL2, 3, 6, 9 or 19) can be enhanced by Th1-related inflammatory mediators including IFNγ, IL-6, TNFα, or LPS [28,29] as well as modulating the effects of each other (Figure 15). The outcomes of such interactions lead to a cascading cytokine pro-inflammatory dysregulation that propagates Gaucher disease. Indeed, TNFα is a major regulator of chemokine gene expression, e.g., CXCL2, CCL3 and IL-6 [30-37]. The finding here that TNFα protein was up regulated (Figure 11) implicates its downstream cytokine network during Gaucher disease progression. Also, cytokines, e.g., CCL3, act synergistically with other macrophage chemokines [38] to maintain the pro-inflammatory reactions.

Pro-inflammatory cytokines play a critical role in macrophage/leukocyte recruitment and adhesion [39-42] and they recruit new macrophages to involved tissues via this cytokine network. Under the stimulus of accumulating glucosylceramide and other glucolipids, such a positive feedback macrophage-cytokine-macrophage cycle can be envisioned to expand and promote progression and the recruitment of additional pro-inflammatory cytokines/mediators networks (Figure 15). The progressive cascade is schematically shown in Figure 15 in which numerous interacting cytokines and chemokines are progressively up-regulated during disease progression from 4 to 28 wk. IFNγ central to this cascading network with initial mRNA up-regulation of β- and α-chemokines, as well as Mrs1 (macrophage scavenger receptor 1), TNFα, and NOS2. Among these interacting factors is the pleiotropic cytokine IL-6, which is a systemic alarm for tissue damage [43-45]. The β-chemokines, CCL2, CCL3, CCL6, CCL9, CCL19, and the α-chemokines, CXCL1, CXCL12, mediate pro-inflammatory effects in the various types of cells and also have synergetic effects on their targets [30,33,46]. The macrophage scavenger receptor 1 (Msr1) has been implicated in many macrophage-associated physiological and pathological processes through endocytosis [47]. NOS2 and arginase were up-regulated at the RNA and protein levels in the 9 V/null mice, and as has been observed in *ex vivo *studies [48]. NOS2 produces nitric oxide from arginine and stimulates pro-inflammation [49-51]. NOS2 production in macrophages up-regulates vascular endothelial growth factor (e.g. VEGF) production and activates angiogenic activity [52,53]. In comparison, arginase2 is a negative angiogenic regulator that inhibits NOS2 activity [54]. Thus, arginase2 and NOS2 alternative pathways in activated macrophages [24] and their up-regulation in 9 V/null mice simultaneous pro- and anti-inflammatory networks are being activated in the Gaucher disease process.

Interrogation of the IL-4 mediated anti-inflammatory network highlights significantly differential expression of 11 anti-inflammatory genes, indicating that participation of the aamφ IL-4 pathway that counteract expression of macrophage pro-inflammatory cytokines and induce molecules that facilitate tolerance, healing and expression of innate immunity receptors, e.g. the scavenger receptor, CD163 [7,55,56]. The IL-4 pathway (Figure 15B) displays the IL-4 time course and interactions over the 4 to 28 wk period. After an initial lag period from 4 to 12 wk, a network of such Th2 response genes [57,58] is up-regulated at the RNA and/or protein levels. This network includes the structurally and functionally related matrix metalloproteinases MMP9/12/19 that are endopeptidases important to remodeling processes [9,17,59,60]. These MMPs are among the most highly-expressed genes in most 9 V/null tissues. The high level expression of MMPs correlated with chronic fibrotic processes in 9 V/null lung and liver (unpublished observation). MMP12 expression occurs in human alveolar macrophages [18,61] and airway smooth muscle cells [62], and in murine alveolar type II epithelial cells [63] or primary lung fibroblasts [64]. The immunohistochemistry showed very strong signals in the lipid-laden macrophages, and much weaker signals in other lung cells (Figure 12). The extremely high level of MMP12 expression (37 to 56-fold in 9 V/null lung and 16 to 22-fold in 9 V/null liver) implicates the aamφ in Gaucher disease progression.

The aamφs are also implicated in the disease progression in 9 V/null mice as evidenced by CD163 expression [10,65] and, particularly, expression of the IL-1 receptor antagonist (IL-1rn). IL-1rn inhibits the activities of IL-1A and IL-1B, and modulates IL-1 related immune and inflammatory responses. IL-1rn is typically produced by aamφ and regulated by IL-4 [66,67]. The interactions of these anti-inflammatory cytokines are schematically shown in Figure 15 and indicate an overall description of pro-/anti-inflammatory networks in *Gba1 *mutant mice.

The global gene expression networks integrate the gene expression patterns observed in 9 V/null or 4L mice. In these networks, more than 1/3 of significantly expressed genes were connected through the cascade interactions of pro- and anti-inflammatory genes. Although the macrophage involvement is major histopathological finding, about 3% of the genome was significantly altered at a molecular level during the development of the disease process. The propagation of the disease clearly depends on a generalized pro- and anti-inflammatory disruption.

Macrophages display marked phenotype heterogeneity *in vitro *and *in vivo*, including the responsiveness to endogenous and exogenous stimuli [8]. Such heterogeneity results in differential phagocytosis or endocytosis, intracellular signaling and gene activation or repression [21,68]. Macrophage heterogeneity was observed in the 9 V/null and 4L models by their differential activation and tissue distribution. Large lipid-laden macrophages (CD68 and F4/80 positive) were mostly observed in the lung of 9 V/null mice. Quantitative immunohistochemistry showed only some cytokines/effectors (i.e., IL-6, NOS2, CCL2, CCL3, CCL9, IL-4, and MMP12) were present in ~50% of lung macrophages (Figure 14). However, IFNγ and Arg2 were present in nearly all of such macrophages. The corresponding RNAs of the cytokine/effectors were also up regulated. The basis for this heterogeneous expression of cytokine proteins in macrophages is unknown, but may be due to the different origins, differentiation sates, or maturation of the macrophage populations.

The genetic background of mice can influence gene expression profiles. Interstrain variations (1-3%) of gene expression profiles have been shown in different brain regions of mouse inbred strains [69-72]. For example, such variation can be observed in the differential susceptibility to a wide range of pathogens [73-76]. Here, 9 V/null and 4L mice had mixed strain backgrounds from three in-bred mouse strains FVB and/or C57BL/6J-129Sv. To evaluate the potential effects of mouse strain background on the expression profiles of macrophage activation genes, the WT data sets from FVB and three inbred mixed strains were used in the analyses. The result showed >50% of significantly expressed macrophage activation genes were shared when either of the two background controls were used. There was 83% concordance in the INFγ- and IL-4- regulated pathway genes. In addition, comparative analyses were conducted with duplicate lung RNA chip data from WT adult mice of three different genetic backgrounds (FVB, C57BL/6J, or 129Sv). The combined WT data generated 790 significantly expressed genes in 9 V/null lung (data not shown). About 60% of significantly expressed macrophage genes were concordant between FVB only or strain-matched WT controls, including >90% of INFγ-/IL4-regulated pathway genes (data not shown). The results showed general agreement of the gene expression profiles from individual WT backgrounds with those from WT controls either pure FVB or strain-matched backgrounds. The results show that macrophage activation genes are a significant functional group in the propagation of Gaucher disease in several genetic backgrounds.

## Conclusions

We demonstrated direct relationships between the degree of tissue glucosylceramides and the gene expression profile alterations. These analyses implicate IFNγ-regulated pro-inflammatory and IL-4-regulated anti-inflammatory networks in differential disease progression with implications for understanding the Gaucher disease course and pathophysiology.

## Methods

### Materials

The following were from commercial sources: RNA Later and TOTALLY RNA kit (Ambion, Austin, TX). Antibody sources are as the follows: Anti-INFγ, CCL2, CCL3, CCL9, CD68, F4/80, IL-4, CD68, and Goat anti-rat-HRP (Serotec, Raleigh, NC). Anti-TNFα (Biosource, Camarillo, CA). Anti-Arg1, Arg2 and CD163 (Santa Cruz, CA). Anti-NOS2 (Chemicon, Temecula, CA), Anti-IL6 (R & D, Minneapolis, MN). Anti-MMP12 (Biomol, Plymouth Meeting, PA). Horse anti-goat-HRP, ABC Vectastain and DAB Substrate Kit (Vector laboratory, Burlingame, CA). Sheep anti-mouse-HRP, Streptavidin-Alexa Fluor 488, Goat anti-rabbit Alexa Flour610, Goat anti-rabbit-HRP (Molecular Probes, Irvine, CA). High Capacity cDNA Archive Kit and SYBR Green PCR Master Mix (Applied Biosystems, Foster City, CA).

### *Gba1 *mutated mice

V394L/V394L homozygote (4L) and D409V/null mice (9 V/null) were generated with mixed genetic backgrounds. 4L mice were 50% of C57BL/6J and129SvEvBrd and 9 V/null mice were 50% of FVB, 25% of C57BL/6J and129SvEvBrd [16]. The wild-type (WT) controls were strain genetic background matched adult mice (50% of FVB, 25% of C57BL/6J and129SvEvBrd,). All mice were housed under pathogen-free conditions in the barrier animal facility and according to IACUC standard procedures at Cincinnati Children's Hospital Research Foundation.

### Glycosphingolipid analyses

Glycosphingolipids in liver and lung from *Gba1 *mutant and WT mice (5 mg tissues) were extracted with chloroform and methanol as described [77]. Glucosylceramide analysis was carried out by ESI-LC-MS/MS using a Waters Quattro Micro API triple quadrupole mass spectrometer (Milford, MA) interfaced with Acquity UPLC system. The ESI-MS/MS was operated in the multiple reaction monitoring mode for monitoring transition pair of the individual protonated parent ions and their common daughter ion m/z 264. Calibration curves were built for C16, C18 and C24:1 β-glucosylceramides using C12 β-glucosylceramide as an internal standard (Matreya, LLC and Avanti Polar lipids, Inc.). The extracted glycosphingolipid samples were suspended in methanol containing internal standard and injected (10 μL) into the LC/MS. The level of total glucosylceramide in the liver and lung were normalized to mg tissue weight.

### RNA preparation and microarray hybridization

Lungs and livers were collected from 9 V/null and 4L mice at the age of 4, 12, 18 and 28 wk, and from genetic background matched adult WT mice (28 wk) and age-matched FVB WT mice (4 - 28 wk) for total RNA preparation. Collected tissues were immediately immersed in RNA Later and tissue RNAs were extracted using the TOTALLY RNA kit. Each tissue RNA set for microarray analysis was pooled from 3 age and genotype matched mice and at least duplicate sample sets for each genotype, tissue, and age were used. This represents 39 labeled RNA sample sets (4 WT, 8 9 V/null and 8 4L lung RNAs; 3 WT, 8 9 V/null and 8 4L liver RNAs) were submitted to the CCHRF Affymetrix Microarray Core for hybridization to Affymetrix GeneChip Mouse Genome 430 2.0 Arrays using standardized protocols. Labeled cRNA synthesis, GeneChip hybridization, washing and staining followed standard Affymetrix protocols. The probed arrays were scanned with the Affymetrix GeneChip^® ^Scanner 3000 and the intensities of array signals were captured with GeneChip Operating Software (GCOS) v1.1.0, according to standard Affymetrix procedures. The entire microarray data set is available at the Gene Expression Omnibus (GEO) accessible through GEO series accession number GSE23408.

### GeneChip quality assessment

Array data were evaluated for quality assessment using the procedures in the Affymetrix based on scaling factor (10-50), percent present (>30%), and housekeeping gene yielding 3'/5' signal ratios <3. Global normalization for outliers or "bad chips" was conducted with each sample/chip. The normalized intensity values were subjected to hierarchical clustering to determine the relative signal intensity of tissue RNA samples derived from different developmental stages and to identify the outlier(s) or bad chips. GeneChips that passed these screenings were used for subsequent analyses to identify significantly affected genes in tissues of 4L and 9 V/null mice at various time points. To evaluate the affects of strain or genetic background, additional control chip data were from age-matched WT FVB mice (4 to 28 wk, 2 chips for each age group).

### Microarray data normalization and analysis

The chip data for each mouse variant and corresponding WT controls with 2 tissue types and 4 different time points were loaded into Partek Genomics Suite 6.4 (Partek, Inc., St Louis, MO) and normalized using the RMA (robust multiarray average) algorithm [78]. Sample relationships were examined using principal components analyses that revealed no strong technical effects, which may encumber the subsequent analyses. To identify expression changes between genotypes, a mixed-model ANOVA was performed and chosen to partition subject, tissue, age, and genotype. The following linear mixed model (equation) was generated to detect differential expression on a gene-by-gene basis:

$${\text{Y}}_{\text{ijkm}}={\text{G}}_{\text{i}}+{\text{T}}_{\text{j}}+{\text{A}}_{\text{k}}+{\text{GTA}}_{\text{ijk}}+{\text{S}}_{\text{m}}+\varepsilon_{\text{ijkm}}$$

Here y_ijkm _is the expression of the gene for ith genotype, jth tissue, and kth age, and mth subject. The symbols G, T, A, GTA and S represent effects due to genotype (G), tissue (T), age (A), genotype-by-tissue-by-age interaction (GTA), and subject (S). The error for each gene for sample ijk is designated ε_ijkm_. Genotype, tissue, and age are fixed effects, and subject is a random effect in the mixed model. For each comparison, a linear contrast was set up to obtain the relative fold changes between each mutant and WT control for each tissue at all time points. Four contrasts were added in the computation: 9 V/null vs. control and 4L vs. control in lung and liver. False Discovery Rate (FDR) was used to further guard against false positives because of multiple testing [79]. FDR was set at ≤0.01 and fold change was set at ±1.8.

### Functional classification

Significantly affected or differentially expressed genes were subjected to an intensive search to identify biological functions. Functional classifications were performed using the Gene Ontology classification obtained through the DAVID Bioinformatics Database (available at http://david.abcc.ncifcrf.gov/home.jsp), and public information and/or literature references. The enriched functional categories were determined by Fisher Exact Test using the corresponding murine genome as a reference dataset. The significance was set at *p*-value < 0.05. The differentially expressed genes were grouped into the following categories (Table 1): catalytic activity, cell death, cytoskeleton, immune response, intracellular signaling cascade, kinase activity, lipid metabolic process, lysosome, macrophage activation, response to stress, transcription and transport.

### Clustering of gene expression profiles

Hierarchical cluster analysis of the significantly expressed genes was performed using GeneSpring GX 7.3 (Agilent Technologies, Inc., Santa Clara, CA), which showed the correlated groups of genes and their expression patterns across all time points.

### Network and Pathway analysis

The significantly differentially expressed genes in the lung of 9 V/null or 4L were loaded into PathwayArchitect 2.0.1 (Stratagene, La Jolla, CA) and built into IFNγ- or IL-4- regulated pathways and global networks. The pathways and networks were constructed based upon the published literature, Ingenuity Pathways Analysis (Ingenuity Systems, Inc), and PathwayArchitect 2.0.1 (Stratagene, La Jolla, CA).

### Common genes selection

The common differentially expressed genes and consistently expressed macrophage activation genes in the lung or liver between 9 V/null and 4L mice were displayed as Venn diagrams.

### Real-time RT-PCR

To verify selected targets from the RNA chip data, real-time RT-PCR assays were developed. RNAs (10 μg) from the same pooled samples as used for microarray chip analyses were used for real-time RT-PCR assays. Each RNA sample was reverse-transcribed (RT) using High Capacity cDNA Archive Kit to synthesize total RNA-cDNA templates with random hexamers. Real-time RT-PCR was conducted using SYBR Green PCR Master Mix with sequence specific primers for CCL9, Msr1, CCL17, MMP12, and β-actin cDNA (Additional file 7 Table S2). The reaction mixtures were incubated in ABI/Prism7000 Sequence Detection System for 40 cycles (95°C, 15 sec and 60°C, 20 sec). The primers were designed with Primer Expression 2.0 (Applied Biosystems) and spanned exon/exon conjunctions (Additional file Table S2). The real-time RT-PCR signals from each RNA primer set were normalized by β-actin signals.

### Histological studies

Mouse tissues (liver and lung) were collected and fixed in 10% buffered formalin for hematoxylin and eosin (H&E) staining and light microscopic studies. For immunohistochemistry and immunofluorescence staining, tissues were fixed in 4% paraformaldehyde/phosphate buffered saline (PBS), pH 7.4, and processed for frozen sections. Tissue sections were blocked with 5% nonfat milk containing 0.4% Triton X-100 in PBS. Tissue sections were incubated with primary anti-mouse cytokine antibodies at 4°C overnight, and then with the compatible secondary antibodies at room temperature (1 h). The secondary antibodies were conjugated with fluorescene or horseradish peroxidase (HRP), or a biotinylated secondary antibody/streptavidin-dye system was used. CD68 (FITC) was used as a macrophage marker, and cytokines were detected using specific antibodies and biotin/streptavidin-conjugated dye Alexa Fluor-610. Positively stained macrophages were quantified using a series of 20 frames (magnification 400×) and counted using MetaMorph 6.1 (Universal Imaging Corp, Downingtown, PA).

## Competing interests

The authors declare that they have no competing interests.

## Authors' contributions

YHX drafted the manuscript, participated in the design the study, and contributed to experiment. LJ contributed to bioinformatic analyses. BQ and MZ contributed to experiment. KS contributed to histological analyses. BA contributed to bioinformatic analyses. YS, WJZ and KDRS contributed to glucosylceramide analyses. GAG initiated, guided the study project, and drafted the manuscript. All authors read and approved the final manuscript.

## Supplementary Material

Additional file 1 **Differentially expressed genes in the lungs and livers of 9 V/null and 4L mice**. Listed significantly differentially expressed genes in the lungs and livers of Gaucher mouse models D409 V/null (9 V/null) and V394L (4L). Click here for file

Additional file 2 **Classification of differentially expressed genes in the lungs and livers of 9 V/null and 4L mice**. Listed functional classification of differentially expressed genes in the lungs and livers of 9 V/null and 4L mice. Click here for file

Additional file 3 **Differentially expressed genes in 9 V/null lungs (cluster 1 in Figure **1**)**. Listed differentially expressed genes and their functional classification in the lungs of 9 V/null mice (cluster 1 in Figure 1). Click here for file

Additional file 4 **Consistently and significantly expressed macrophage activation genes in 9 V/null and 4L tissues**. Listed up-/down-regulated and consistently expressed macrophage activation genes in the lungs and livers of 9 V/null and 4L mice. Click here for file

Additional file 5 **Common and unique macrophage activation genes in 9 V/null and 4L tissues**. Listed common and unique macrophage activation genes in the lungs or livers between 9 V/null and 4L mice. Click here for file

Additional file 6 **Differentially expressed genes in 9 V/null lung vs. WT with various backgrounds**. Listed differentially expressed global genes and macrophage activation genes and their commonality in 9 V/null lungs vs. WT with various backgrounds. Click here for file

Additional file 7 **Table S1**. The effect of WT mouse strain background on the gene expression profiles Analysis of altered genes in 9 V/null lung against the different wild type (WT) backgrounds: FVB/129Sv/C57BL6J mix or FVB WT. Table S2. Primers for real-time RT-PCR. Listed the primers for real-time RT-PCR analyses. Click here for file

Additional file 8 **Differentially expressed genes in network in 9 V/null and 4L lungs**. Listed differentially expressed genes in network and their functional classification in 9 V/null and 4L lungs. Click here for file
