# Supplementary material for: Global gene expression profile progression in Gaucher disease mouse models
Source: BMC Genomics. 2011 Jan 11;12:20. doi: 10.1186/1471-2164-12-20 (PMC3032697; doi:10.1186/1471-2164-12-20)
Supplement: Additional file 7 — Table S1. The effect of WT mouse strain background on the gene expression profiles Analysis of altered genes in 9 V/null lung against the different wild type (WT) backgrounds: FVB/129Sv/C57BL6J mix or FVB WT. Table S2. Primers for real-time RT-PCR. Listed the primers for real-time RT-PCR analyses. [file 1471-2164-12-20-S7.DOC]

Additional file 7, Table S1. The effect of WT mouse strain background on the gene expression profiles

|  | FVB/129Sv/C57BL6J (28 wk) | FVB (4-28 wk) |
| --- | --- | --- |
| # of significantly expressed genes | 1171 | 910 |
| # of macrophage activation genes | 124 | 90 |
| # of pathway genes | 18 | 15 |
| FDR | <0.01 | <0.01 |

The numbers of genes that were altered in 9V/null lung, which were analyzed against the different wild type (WT) backgrounds. FVB/129Sv/C57BL6J: the matched WT background with 9V/null mice. FVB: FVB background WT samples at 4 different time points.

Additional file 7 Table S2. Table 2. Primers for real-time RT-PCR

| RNA | 5’-primer from | Sequence | Accession |
| --- | --- | --- | --- |
| CCL9 | 5’-primer from 171st bp | 5’-TGCCTCTCCTTCCTCATTCT-3’ | NM_011338 |
|  | 3’-primer from 242nd bp | 5’-TTTGTCTCTGTTGCATGTGTGATC-3’ |  |
|  |  |  |  |
| CCL17 | 5’-primer from 31st bp | 5’-ACCATGAGGTCACTTCAGAGCT-3’ | NM_011332 |
|  | 3’-primer from 159th bp | 5’- AATGGCCCCTTTGAAGTAATCC-3’ |  |
|  |  |  |  |
| Msr1 | 5’-primer from 109th bp | 5’-CATCCCTTCCTCACAGCACTAAA-3’ | NM_031195 |
|  | 3’-primer from 201st bp | 5’-ACACAAGGAGGTAGAGAGCAATGA-3’ |  |
|  |  |  |  |
| MMP12 | 5’-primer from 352nd bp | 5’- ATGAAGCGTGAGGATGTAGACTACA-3’ | NM_008605 |
|  | 3’-primer from 493rd bp | 5’- TGAAGTCTCCGTGAGCTCCAA-3’ |  |
|  |  |  |  |
| β-actin | 5’-primer from 419th bp | 5’-GGCCAACCGTGAAAAGATGA-3’ | NM_007393 |
|  | 3’-primer from 497th bp | 5’-CACAGCCTGGATGGCTACGT-3’ |  |
